# Location based bursty event detection and information dissemination using influencers in Twitter

**DOI:** 10.1038/s41598-026-44512-6

**Published:** 2026-04-29

**Authors:** D. Manikandan, C. Valliyammai

**Affiliations:** Department of Computer Science and Engineering, College of Engineering Guindy, Anna University, Chennai, India

**Keywords:** Machine learning, Deep learning, Social media, Event detection, Community detection, Influencer identification, Engineering, Natural hazards

## Abstract

In the intelligence information sharing era, social media paved a way to share information around the world without any moratorium, which helps more in times of crisis. Influencers are a central node who play an important role in information diffusion throughout the community. Identifying the influencer and predicting the geospatial location of the bursty event during a crisis are the most challenging task in an emergency. The proposed location based bursty event detection and influencer identification helps to send the emergency alert message of disasters to the communities through the influencer at the right time. The traditional machine learning models and Bidirectional Encoder Representations from Transformer model are trained to detect emergency event tweets, in which the Valence Aware Dictionary for Sentiment Reasoning + Count Vector + Ensemble model outperforms well with 98% accuracy. The location of the tweets is extracted by a location tagger and Name Entity Recognizer using Natural Language Processing. The Louvain based Harmonic Centrality Algorithm is used to detect user communities based on the location of users mentioned in the tweets, where the community of the network obtains a modularity of 0.75. The integration of harmonic centrality algorithm identifies the influencers of each community in the network which helps in reducing the disaster risk at the time of emergency as well as early dissemination of information throughout the network community.

## Introduction

Social Network leads a major role in diffusing information throughout the communities and it provides an open and user-friendly platform to share and communicate information with individual friends, groups, organizations, companies, etc.^1,2^, through online using smart devices in which it helps to capture the information of the user and their posts, behavior, replies or retweets, emotions and opinions of the significant user^3^. Disasters are unpredictable but it is common in day to day life and it affects huge area and mass population of human where the events are identified by analysis of geo-tagged data, disaster distribution, and disaster occurrence frequency^4^. The sub events detection on social media was proposed to detect the sub-events by filtering and enrichment process of user posts shared in social media^5^. Social media paved the way for the crowdsourcing concept in which multiple user’s share their view, ideas which helps in making a right decision at right time^6^. The role of crowd-sourcing was analyzed for the wildfire case study of a Croatian city in which it helps to share awareness among the crowd about the current situation^7^. Social media applications are more useful in the time of emergency for finding volunteers^8^ and for raising funds to safeguard the people affected by disaster.

Twitter is one of the fast growing microblogging social media services and it has emerged as a popular communication channel to collect and diffuse breaking news in a conscientious time^9^. Twitter social media are more active at the time of crisis^10^ and it leads the major role in disseminating information^11^ and making significant bursty disaster topics to be more trend with less time duration^12^ when compared to other social media. The diffusion of information in social media faces major challenges such as choosing the right influencer to share the information^13^ throughout the network by believing the trustworthiness of certain people such as politicians, actors, actresses, trending You Tubers, etc. Choosing the right time is the other biggest challenge because many communities in social media will be active at a significant time. Choosing the right audience is also a challenge in social media to spread information throughout the network^14^. These challenges are overcome at the time of crisis based on one significant user post; the information is diffused throughout the communities by influencers. The influencers in the social media network play an extraordinary role in sharing the right information at the right time. Influencers should possess certain conditions by choosing the right audience and should be active at the right time, credible and expert to share the information and should be a great trendsetter^15^. The influencers should also possess some of the characteristics such as being authenticated and trustworthy people^16^. The followers in the network also lead a great role in sharing information with different communities.

The crisis events are unpredictable such as natural disasters and it causes more damage to human life^17^. Social media contributes more, at the time of disaster by taking the complex decisions and effective actions faster to save the life of humans. Influencer is an authorized person who helps to collect, share and spread the information throughout the network by detecting community based on the location. By sharing the emergency alert messages of the specified location helps the people to prevent their life and families from the disaster^18^ motivated us to propose a location based bursty event detection and information dissemination using influencer in Twitter.

### Related works

Disaster tweets in social media are closely related to the sentiments of the user who post the information. Event detection helps to identify the location of disaster using geo-tagged tweets. Community detection helps to spread the information to a large network with the help of influencers in the network.

#### Sentimental analysis using social media

Twitter social media offers a strong background for sentiment analysis in which it plays a crucial role in identifying the sentiments and emotions of the message^19^. Sentiments have great power in social media to express the user’s feelings^20^. Lexicon based approach^21^ was implemented to analyze the sentiment from a dictionary or linguistic data. An efficient algorithm for sentiment analysis^22^ was used to detect the signals on Twitter based on the volume of tweets, hashtags, and sentiment. Twitter sentiment analysis was also used in preprocessing technique^23^ on e-learning decision support systems. The sentiment analysis of disaster events helps to identify the emotions and sentiments of the people^24^ who suffer in the disaster which also creates situation awareness among the people^25^. The geospatial sentiment analysis^26^ was performed with multimedia disaster data in which sentiment was extracted by aggregating geographical-based local region insights. Emotions are the greatest weapon to express the current situation of the users. More emotions are shared at the time of emergency events in which emotions differ from person to person. The classification of emotions was done to estimate the different reactions from the user to a significant post and it was used to give the alert message and to make the right decision at the right time. The sentiment analysis was more useful at the time of pandemics and natural hazard crises^27^ in which it helps to find the sentiment of the user and provide relief measures. The sentiment classifiers^28^ were used to categorize the emotions of the media users at the time of disaster. The VADER sentiment analysis^29^ was used in social media to detect the sentiment, emotions, and behavior of the user in which it works under a parsimonious rule-based model where sentiments are analyzed based on the valence and polarity score. The hybrid-based sentiment analysis^30^ was introduced which comprises of three different models such as LSTM model, word embedding, and VADER sentiment analysis model where these models are integrated to produce a target value in binary numbers.

#### Event detection using social media

Social media helps in sharing emergency events^31^ with the help of the Bi-LSTM deep learning model, where events are detected based on the three phases’ similarities, such as type of events, location, and time, by clustering mechanisms. Event classification was the first phase to detect the event by removing irrelevant data, while location extraction and time extraction were the second and third phases, respectively. The seed method was used to collect the multiple posts that are relevant to the event. The shannon entropy^32^ was introduced to detect events with a specific location by improving the accuracy which helps to identify the user information, time interval, and location of the event. The entropy, event detection hit score, and event cluster score are some of the parameters used to identify the event location. The latent dirichlet allocation and density-based clustering methods^33^ are integrated to detect and track event information from social media, where geo-tagged tweets are extracted based on the temporal data in which the tweets are identified based on topics and clustered. An information gain and chi-square were used to select the features to classify the text^34^. The BERT-Att-Bi-LSTM model^35^ was introduced to detect emergency events based on the user’s posts on social media. An unsupervised dynamic clustering algorithm was used to cluster the similar text of the post. The logistic regression, a supervised learning model, was used to cluster the post by categorizing different events. A three-phase framework such as classification, extraction, and clustering was designed to extract the information from the three W attributes such as what, where, and when. The Distil-Bert and non-meta-heuristic techniques^36^ are integrated to extract the relevant features from the crisis dataset, whereas hunger game search is a non-heuristic technique to remove the irrelevant features from the extracted features. A combination of Convolution Neural Network (CNN) and Bidirectional Long Short Transformer (Bi-LSTM) helps^37^ to detect the geographical-based location detection from Twitter. The stochastic gradient descent method was used for training the model for predicting tweet text, user profile location, and screen name. A five-fold cross-validation technique was used to improve the efficiency.

#### Community detection using social media

Social Media Network is massive, and each node is interconnected to the other, so different users may have different opinions on the different domains. The different domain users are grouped together to form a community in which the user can share their own views, comments, and replies to their posts. The bridge nodes and central nodes are identified based on the centrality where as a community detection algorithm^38^ was introduced to identify the independent features in the network. The weighted label propagation Algorithm^39^ was implemented to detect the community with the help of node importance and a weighted link connected with the other node, where the algorithm introduced a formula for the node by weight updation based on the node- link and importance. An autonomous divisive algorithm^40^ was introduced to detect the communities based on the link breakage strategy, which helps in removing weak links and link breaks where it was identified based on linked nodes connected in the boundary of the community. The betweenness centrality was implemented to calculate the removal of links continuously. The delphi method^41^ was introduced to identify the influencers in social media based on the community in which two rounds were performed to find the influencer; the first round was introduced to categorize an open questionnaire, where the second round was used to achieve consensus based on the influencer in the network. The community of large-scale network was detected using a graph where the faster louvain algorithm^42^ was introduced to detect the community in which it undergoes two processes such as dynamic iteration to detect the communities where splitting the local tree structure was the second process to optimize the efficiency of the algorithm through the number of iterations. Three- stage algorithms^43^ were introduced to detect the community in the social media where the central node was identified based on the surroundings of the neighbor in the network. The label propagation was the second algorithm to label the node based on the color where the same color represents the same community. The combination of the communities with maximum similarities was the third algorithm where many communities are merged to form a single community. The label Propagation Algorithm was used to detect the communities based on the directed signed of social networks^44^. The graph convolution neural network was used to detect the unsupervised community in social media^45^. The community was detected based on the social behavior^46^ of the user in the network where the louvain algorithm^47^ and novel cyber-attack detection helps to identify the bullies^48^ and girvan newman algorithm was used to detect the hate content in social media^49^.

#### Influencer identification using social media

The influencers in social media perform more activities in the network by sharing useful information at the right time in the right situations. Influencer possesses the trustability features in which it gets identified by using their behavior and activity. In egocentric online social networks^50^ an influential nodes are identified using various analyses such as behavioral activity, network structure, and collaborative analysis in which ego and socio-centric approach help to check whether an influencer belongs to the same network. The graph-based approach^51^ was introduced to identify the influential node in the network by ensemble algorithm to rank the node where the topological approach helped to identify the most influential node based on the location. Influencers are also more popular in the field of marketing, education, medicine, etc. The market marven^52^ methodology was introduced to spread the information based on services, products and motivation ideas and campaigns, etc. Delphi method was introduced to identify the influencer in the field of marketing, management, and organization where two rounds are performed to filter other users from the network. A multidimensional social influencer framework^53^ was designed based on the topic level, global level, and on the exploration of the factors based on the information, action, and structure. Influencers are identified by using topics by fetching the text in dynamic social networks. The link prediction algorithm^54^ was used to improve the accuracy where the probing-based algorithm was introduced to maintain the partial copy of the new user data which helps to identify the influencer. In a complex network, the identification of the influencer of influencer is difficult where edge-based weight updation method was introduced to identify the influencer node. The edge updation method^55^ was introduced to rank the most preferred node in the network where page rank algorithm was implemented to extract the information based on the updated weight. The graphical analysis^56^ helps in identifying the influential nodes in the network where influencers are identified using centrality algorithms such as degree, closeness, eigenvector, betweenness, page rank, etc. The multilayer network model^57^ was implemented to identify the influencer in the social network by using INF centrality which predicts the similar values of the betweenness centrality of the node in the network. SIR model was implemented which helps to compare the rank of the influencer. The principal component centrality^58^ concept was implemented to identify the influential node based on the position of the network in which it identifies the neighbor of the influencer rather than influencer. The graph- based long short-term memory^59^ and charismatic transmission technique^60^ were used to maximize the influencer in the network.

Based on the several studies we have explored social media based influencer identification during crisis are limited by noisy and unstructured user generated content, weak sentiment discrimination, insufficient location specification and improper influencer identification. These limitations often lead to inaccurate identification of bursty events and delayed information dissemination. The several recent approaches rely heavily on deep learning model that demands large labelled datasets and requires high computational resources. In addition, identifying influencer’s user with respect to the location for diffusing information to the right communities at the right time remains the challenging task. These challenges motivate us the need for computationally efficient, interoperable location aware system to detect the crisis events and facilitate timely information dissemination. From these gaps, we proposed a location based bursty event detection and information diffusion using influencers system to enhance the situational awareness to safeguard the users during crisis.

The key contribution of the proposed work follows:

- A new solution is proposed to identify the influencer at the time of disaster by analyzing the user social relationship among geo-tagged information extracted from Assam flood tweets.
- The vader sentiment analysis technique is used to label the twitter data by capturing an accurate sentiments, emotions and intensity of the tweets which combines with the ensemble classifier, for an efficient handling of unlabeled and noisy data.
- An extensive comparative analysis of traditional machine learning and deep learning model (BERT) are performed to evaluate classification effectiveness and demonstration which improves the performance with low computational complexity
- A location extraction and mapping strategy is developed by integration of geo-tagged data, named entity recognition, and pincode-based location matching to accurately identify bursty event locations.
- A Louvain-based Harmonic Centrality Algorithm (LHCA) is introduced to detect communities and identify influential users, enabling effective and targeted dissemination of emergency information across social media networks.

## Methodology

Twitter is one of the social media, plays a major role in disseminating information where tweets are categorized into many types and the system focuses on geo-tagged tweets, timestamp tweets, and hashtag tweets. All these tweets are extracted from the Twitter API and collected as a dataset. The dataset contains more amount of data which comprise of both disaster and non- disaster tweets. The Tweet data contains more noise and irrelevant data, to overcome this data cleaning process is involved where the tweets obtained from Twitter will not have the label. To label the data into disaster and non-disaster classes, sentiment analysis is performed. The sentiment identification of tweets performs various processes in which each token is analyzed using the VADER lexicon which comprises more data and gets categorized into positive, negative, and neutral based on the polarity score. The threshold value is fixed to label the data depending upon sentiments where tweets are labeled as disaster and non-disaster.

A dataset is enormous in that it comprises more tweets, to classify the crises from the dataset the event classification is performed. The count vectorizer is used to extract the feature from the tweet text into vectors. BERT model uses token, sentence, and position embedding’s to extract the features of the tweets then the data is trained where machine learning and deep learning models are used to classify the data. Based on the performance metrics the best model is opted. After performing classification, disaster tweets are collected separately where the location mentioned for the disaster event is extracted in which a geo-spatial location may be wrong while tagging the post or the user may be in a different location when posting the information about the different location in which it misleads the user at the time of crisis.

**Fig. 1:**
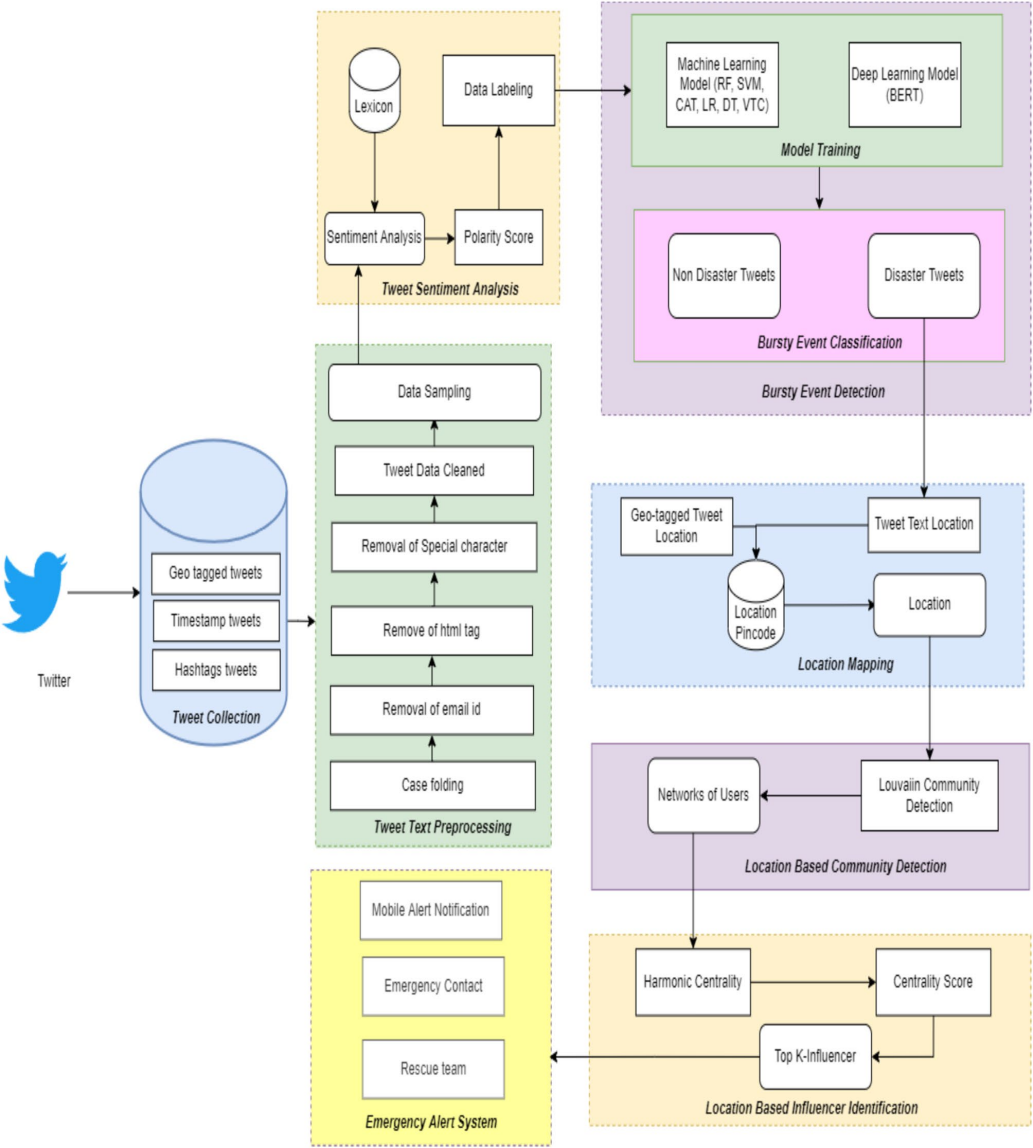
Proposed architecture for location based Bursty event detection and influencer identification using social media.

To overcome this problem location prediction is performed where location is extracted from the tweet text. The geo-tagged location and extracted location from the tweet text are merged into a single dataset where the new database is created with both location and pincode of the specified area is identified using different categories of the tweet. The location dataset and pincode dataset is compared using Structure Query Language (SQL) where a similar location with the pincode is extracted as the new dataset. Based on the location pincode, community is detected using the Louvain algorithm in which user communication is represented graphically using nodes and edges. The communities are detected using user communication at the time of disaster. The influencers are the central node identified in the communities who play a responsible role in sharing information throughout the network. The centrality algorithm such as harmonic centrality is used, based on the ranking scores influencers are identified for the communities. Once disaster information is passed and verified, then the influencer shares the information through his/her community and then it diffuses to other communities and the whole network where the proposed location based bursty event detection and influencer identification system gives the alert message to the user about the disaster and location of the event to the members of the community as shown in Fig. 1 and this helps the rescue department to take steps further to save the people from the disaster.

### Data collection and pre-processing

The data is extracted using the Twitter API using the consumer secret key in which the data are scrapped using significant hashtags such as # 2022 Assam floods. In this paper, we significantly focused on the bursty events such as natural calamities where the recent natural disasters such as the 2022 Assam floods dataset is chosen. Before data is extracted, the required features for the data need to be selected in twitter such as created at, tweet id, receiver name, receiver id, tweet text, profile name, name, account creation date, followers, friends, retweets, and hashtags. Once the data is extracted successfully, a significant or dependent feature is selected to identify the required output. Tweet text is chosen as the significant feature, where data preprocessing technique is used to clean the noisy data in the most efficient manner such as by translating uppercase letters into lowercase letters, removal of data that comprises links, special characters, accent characters, email id, etc. The data removal is done for the blank rows and missing values are filled and then the text undergoes stop words processes in which it is represented using a word cloud where the lemmatization is the next step to frame it as a meaningful word. After the noise is removed, the data is ready for the labeling process.

### Sentimental analysis and data labeling

The real-time tweets of the Assam floods are collected, so the dataset is unlabeled, to convert the unsupervised learning model into the supervised model, a data labeling is required in which the flood dataset is unlabeled data. VADER (Valence Aware Dictionary sEntiment Reasoner). A sentiment analysis uses Natural Language ToolKit (NLTK) to label the data. The preprocessed data are compared with the VADER lexicon or dictionary is a rule-based system containing more than 7500 lexicon text that helps to identify the sentiments. Tweets are well analyzed and it gets categorized into different sentiments such as positive, negative, and neutral. The polarity score of tweets is more dependent to detect the sentiments where the scores are generated for all three sentiments and it mostly depends on the intensity of the tweets. The compound score is another parameter that helps to detect whether the tweet is positive or negative and it is calculated by summing the valence score of each word in the tweets with the VADER lexicon.

1 $${\mathrm{Tweet}}\,{\mathrm{Compound}}\,{\mathrm{Score}}\, = \,({\mathrm{VS}}[{\mathrm{w1}}\left] { + {\mathrm{VS}}} \right[{\mathrm{w2}}\left] { + \cdots + {\mathrm{VS}}} \right[{\mathrm{wn}}])$$

The polarity of a compound score of the tweet depends on all three sentiments, by choosing the high sentiment score value. The positive polarity score of the significant tweets is higher when compared to negative and neutral polarity score then the compound score value is positive, as the same negative polarity score of the tweet is higher when compared to positive and neutral then the compound score is considered to be negative. The compound score is neural when it doesn’t reach the above scenario.

2 $${\mathrm{Tweet}}\,\,{\mathrm{Compound}}\,\,{\mathrm{score}} = \left\{ {\begin{array}{*{20}l} {Positi,} \hfill & {Polarity(Positive) > Polarity\,(Negative)} \hfill \\ {Negative,} \hfill & {Polarity(Positive) < Polarity\,(Negative)} \hfill \\ {Neutral,} \hfill & {Otherwise} \hfill \\ \end{array} } \right.$$

To detect the accurate disaster-related tweet data threshold value ‘z’ is fixed. Sentiments of the tweets are calculated with the threshold value and compound score of the tweet. Positive sentiment reveals that the compound score should be high when compared to a threshold value. Negative sentiment relates that the compound score should be less than the threshold value.

3 $${\mathrm{Sentiment}}\,\,{\mathrm{of}}\,\,{\mathrm{tweets}} = \left\{ {\begin{array}{*{20}l} {{\mathrm{Positive}}\,{\mathrm{Sentiment}}} \hfill & {CS > Z} \hfill \\ {{\mathrm{Negative}}\,{\mathrm{Sentiment}}} \hfill & {CS < Z} \hfill \\ \end{array} } \right.$$

### Event detection and data sampling

After Positive sentiment tweets are labeled as ‘1’ and the negative sentiment tweets are labeled as ‘0’ where the dataset comprises of a large volume of data and it is very difficult to classify the disaster and non-disaster tweets. The traditional machine learning model such as Random Forest (RF), Decision Tree (DT), Support Vector Classifier (SVC), Naïve Bayes (NB), Multinomial Naïve Bayes (MNB), Extreme Gradient Boosting Classifier (XGB), Logistic Regression (LR), Cat Boosting Classifier (CBC), and Ensemble Classifier (LR + NB+ XGB) are trained to classify the tweet. Machine learning model performs with the Assam flood dataset, Deep learning model is capable to train the large dataset to get the accurate result because the model takes more hidden layer and it doesn’t require feature engineering. Bidirectional Encoder Representation from Transformer (BERT) is a traditional Recurrent Neural Network (RNN) is used to classify the disaster data.

The BERT model is implemented to understand the context and language of the data in which the model comprises two segments such as Masked Language Modeling (MLM) and Next Sentence Prediction (NSP). The BERT model performs the data encoding and transformation of data, the tokenization is the first process in which the sentence is converted into tokens one by one where an autoregressive language model such as GPT 3 helps to produce the text like human- made and it is masked using the upcoming token. CLS and SEP are the special tokens added to separate the words where CLS token helps to classify the sentences and it is added at the beginning. SEP helps to separate the sentences for predicting the next sentences.

4 $${\mathrm{TBERT}} = < {\mathrm{TCLS}},{\mathrm{T1}},{\mathrm{T2}},{\mathrm{T3}}, \ldots {\mathrm{TSEP}} >$$

The separated tokens are embedded using token embeddings where these tokens are grouped together as segments which start from the CLS to SEP for the first sentence and SEP to SEP for the next sentences and it goes on. The padding is performed to add the empty tokens when two concatenated sentences are smaller than the maximum word length. The truncation is performed to cut short the sentence which exceeds the maximum word length which helps to truncate the long sequence. Attention mask helps to send a batch of words into the transformer which pads all sentences of the same length.

5 $${\mathrm{E}}[{\mathrm{TBERT}}\left] { = < {\mathrm{E}}} \right[{\mathrm{TCLS}}],{\mathrm{E}}[{\mathrm{T1}}\left] {,{\mathrm{E}}} \right[{\mathrm{T2}}\left] {, \ldots {\mathrm{E}}} \right[{\mathrm{TSEP}}] >$$

The position embedding is also performed for each and every token and it finally forms encoded data in which the tokens are referred using individual encoded data or id’s.

6 $${\mathrm{E}}[{\mathrm{TBERT}}] = < {\mathrm{E}}\left[ {1} \right],{\mathrm{E}}\left[ {2} \right],{\mathrm{E}}\left[ {3} \right], \ldots {\mathrm{E}}\left[ {\mathrm{n}} \right] >$$

The BERT model is trained with English language and it is masked using masked language modeling where 15% of the words is randomly masked in which it helps to predict the word. The BERT allows the model to train and learn the sentence bi-directionally. The NSP concept is implemented to predict the upcoming sentence from the significant sentence where the sentences are already masked and the concatenation operation is performed with masked sentences and it is taken as the input for the model. The NSP helps to identify the sentence next to the trained sentence where the BERT model helps to predict that two sentences are interrelated to each other by learning the inner representation of English language in the sentences. The model takes the embedded tokens as input and it moves towards the hidden layer in which dense layer is a deeply connected neural network where the dropout layer drops the connection between the networks to avoid overfitting. The softmax layer helps to calculate the probability of data to fit for every possible class.

After training the data in the dataset, learning models are used to classify the data, due to data imbalancement, it leads to misclassification. To overcome this issue, a sampling technique is performed to balance the dataset. The oversampling concept helps to equalize the lower count data to higher count data. The downsampling technique is also used to equalize the high-count data to low-count data for processing the large data fast.

### Location collection and mapping

The geo-tagged locations are extracted to identify the disaster-related locations where in many cases, a user in the social media network doesn’t tags the location and it is difficult to identify the disaster at the right time. In many other cases, a user tries to mention the location in the tweets without tagging where these tweets are not mostly considered but these tweets help to extract the disaster location and to prevent the people. Location tagger and NER library files in python are used to extract the location information from the tweet text. The locations are identified using LOC and GPE entities where their entities help to identify the regions, cities and countries from the tweet text.

The geo-tagged tweets and location extracted tweets are concatenated. The locations pincode database is created with specific features such as location name and pincode. The location extracted database and location pincode database are connected with a similar feature location. A new dataset is created based on Structured Query Language (SQL) using a join clause with a single feature, location and it is mapped where this dataset helps to extract the pincode location of the user tweeted on Twitter.

### Community detection and influencer identification

The community is a group of interconnected nodes that shares similar interest and opinion in social media network and it plays a predominant role in identifying the influencer. Louvain community algorithm is introduced to detect the community for the large network in which it undergoes two different phases such as modularity optimization and community aggregation.

**Figure:**
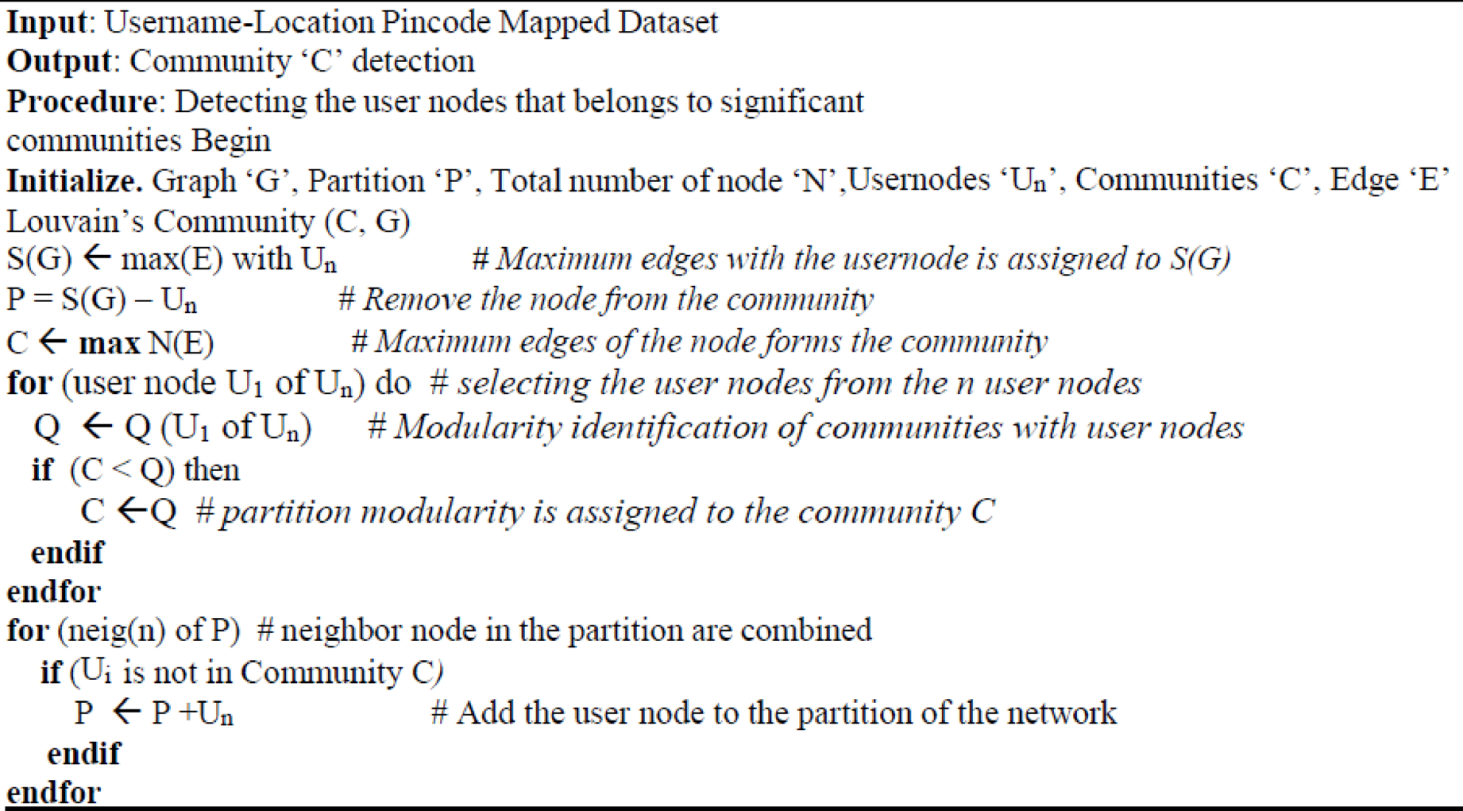
Louvains Algorithm for Community Detection

The louvain algorithm starts with the single node, to check whether the node belongs to the communities or not. A node is randomly selected and it moves from one community to another, to identify the partition of the network. Based on these partitions, the new network is aggregated to form another community and it is continuous and this improves the quality of the network.

7 $$Q = \frac{1}{{2{\mathrm{E}}}}\sum\limits_{x = 1}^{n} {\sum\limits_{y = 1}^{n} {\sum\limits_{y = 1}^{n} {\left( {A_{xy} - \frac{{Dg_{x} \times Dg_{y} }}{{2{\mathrm{E}}}}} \right)} } }$$

‘Q’ represents the modularity of the network; ‘E’ represents the number of edges in the network where *Axy* matrix represents the adjacent matrix with nodes connected with other nodes. ‘x’, ’y’ represents the nodes in the community. Dgx is the degree of node ‘x’ in the network where Dgy is the degree of the node ‘y’ in the network for specified community where ‘n’ is the node in the network.

The degree of the node in the network is calculated by number of the edges related to the vertices where ‘x’ and ‘y’ act as nodes which specifies whether the current node has a link with other nodes or not. Assign x =1, y =1, and the position of the node is identified through the matrix.

8 $$Dg_{x} = \sum\limits_{x = 1,\,y = 1}^{n} {({\mathrm{A}}_{x} )}$$

The degree ‘Dgx’ is identified by adding edge with respect to the corresponding vertex and it continuous for the entire vertex in the network. The aggregation of network is done with assigned node. Depends on the community, ‘Dgy’ is identified by obtaining ‘Dgx’ where ‘Dgy’ is the combination of all ‘Dgx’ for all communities.

9 $$Dg_{y} = \sum\limits_{x = 1}^{n} {({\mathrm{Dg}}_{x} )}$$

10 $$Dg_{y} = Dg_{1} + Dg_{2} + Dg_{3} + Dg_{4} \cdots + Dg_{n}$$

11 $$Q = \frac{1}{{2{\mathrm{E}}}}\left( {A_{xy} - \frac{{Dg_{x} \times \left( {Dg_{1} + Dg_{2} + Dg_{3} + Dg_{4} \cdots + Dg_{n} } \right)}}{{2{\mathrm{E}}}}} \right)$$

After detecting the communities, an influencer is identified using the centrality score of the node. In Graph based analysis, Harmonic centrality plays a vital role in identifying influencer nodes in the network. The centrality is identified based neighborhood node and path connected with the specified node in the network. The centrality algorithm helps to identify the influencers in both small and large communities. The harmonic centrality works similarly to closeness centrality, which helps to find the shortest path with the nodes and it is also used to solve the unconnected graph. The geodesic distance matrix is used to identify the shortest path with a significant node with the other nodes in the network. The link score (Ls) is calculated by summing the reciprocal of the shortest path distance of a significant node with other nodes and it follows for other remaining nodes. The harmonic centrality (Hm) is calculated with the ratio of the link score to the community order (m1) for the given network.

12 $${\mathrm{L}}_{{\mathrm{s}}} ({\mathrm{A}}) = \sum\limits_{{{\mathrm{x}} \pm {\mathrm{A}}}}^{n} {(1/{\mathrm{dis}}({\mathrm{A,}}\,{\mathrm{x}}))}$$

13 $${\mathrm{H}}_{{\mathrm{m}}} ({\mathrm{A}}) = {\mathrm{L}}_{{\mathrm{s}}} ({\mathrm{A}})/{\mathrm{m}} - 1$$

**Figure:**
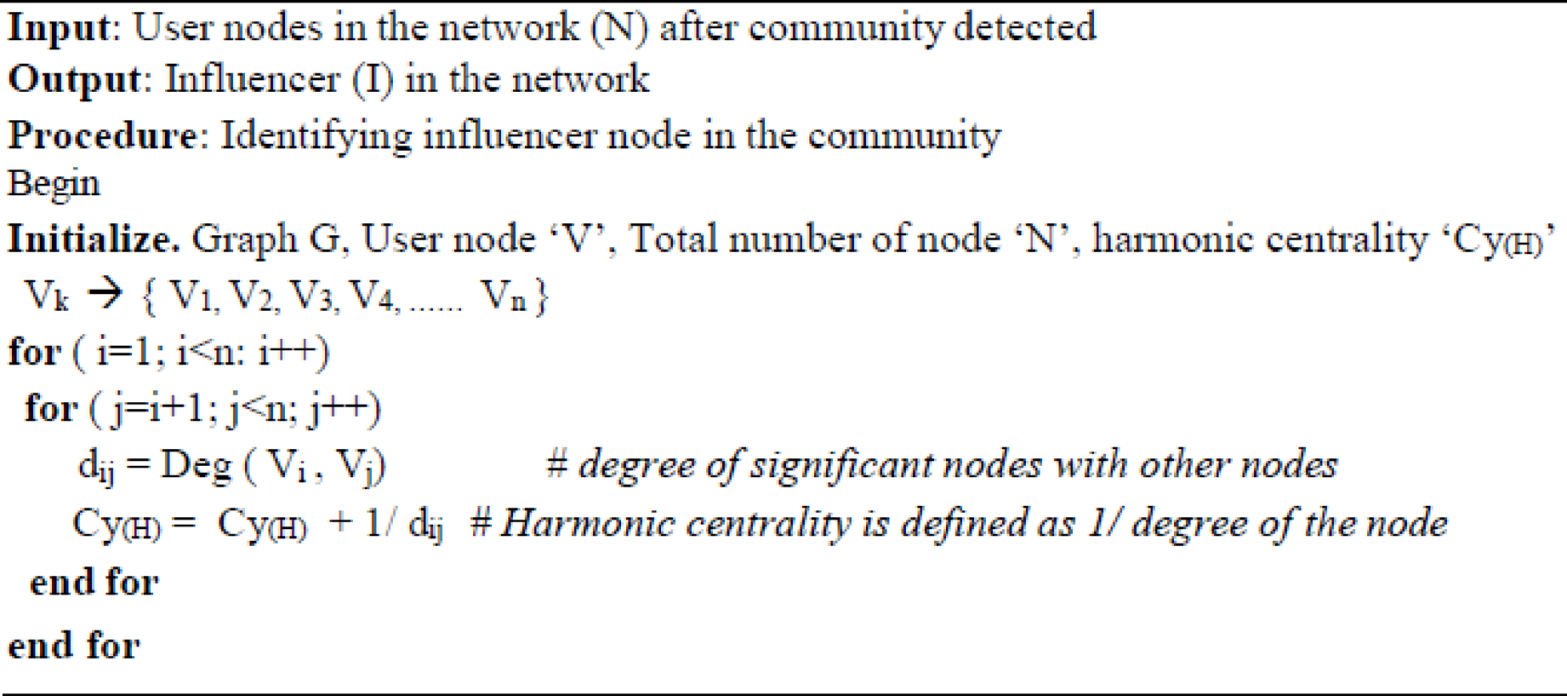
Harmonic Centrality Algorithm for Influencer Identification

## Experimental result analysis

### Dataset analysis

**Fig. 2:**
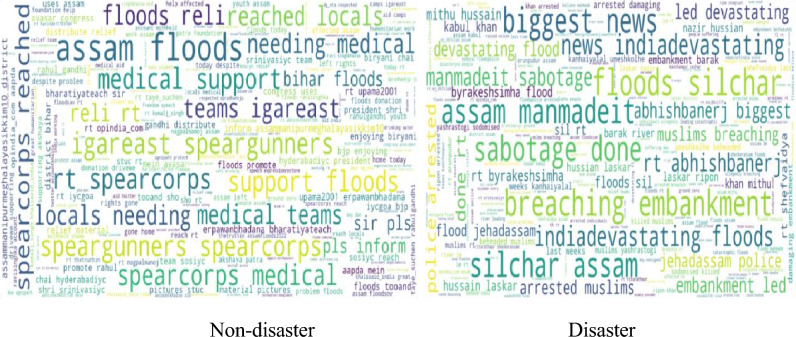
Word clouds of Assam flood 2022 dataset.

A new corpus comprises of Assam flood tweets extracted using hashtag # 2022 Assam Floods. The dataset consists of timestamps, tweet id, mentions, tweet text, locations, user name, hashtags, and user mentions, and retweet counts. The tweet text is selected as a significant feature to identify the disaster in which it undergoes some preprocessing stages to remove the noise in the dataset; the text hero library file is imported for automatic case fold conversion, removal of emails, html tags, special characters, and accented character. The dataset analysis of word cloud representation is shown in Fig. 2.

**Fig. 3:**
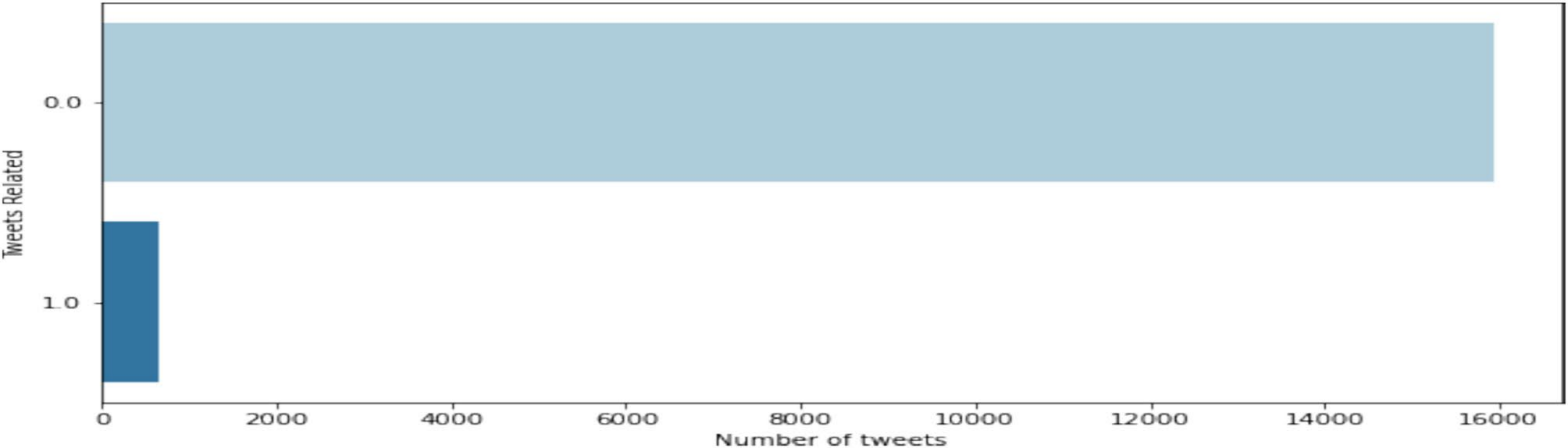
Data labeling in Assam flood 2022 dataset.

Assam Flood Dataset consists of 20,000 tweets collected from Twitter API. The vader sentimental analysis is performed with respect to tweets to label the data which helps to identify the sentiment and intensity of tweets where threshold value ‘z’ is fixed as 0.5, because in Vader all negative lexicons comes with high compound score above 0.6 whereas all positive lexicons comes with low compound score below 0.5 where neutral value is determined as the threshold value. After the sentiment is identified, 655 tweets with positive 3422 neutral sentiments, 15,923 negative tweets based on the compound sentimental score of the tweets are captured. Neutral sentimental tweets are removed and 16,578 tweets are processed for the next stage where positive tweets are labeled with ‘1’ and negative tweets are labeled with ‘0’ as shown the Fig. 3.

**Fig. 4:**
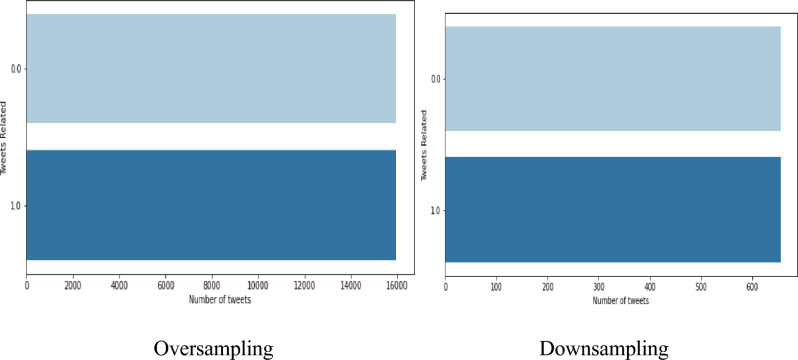
Data sampling in Assam flood 2022 dataset.

The count vectorizer method is implemented to extract the feature in which it converts the textual data of tweets into numerical vectors by tokenizing the data where the vocabulary text data are created and it is encoded as a vector in which it is represented in the spare matrix. The training the data with imbalancement leads to misclassification and to overcome this problem, a sampling technique is introduced to balance the dataset where the oversampling is performed to increase the lower minority class tweets to the higher majority class. Downsampling is performed to decrease the higher majority class tweet into the low minority class. To address the severe class imbalance, Random Oversampling is performed on the minority disaster class. This resulted in an equal distribution of 15,923 tweets per class, yielding a balanced dataset of 31,846 tweets for subsequent analysis and it results is shown in the Fig. 4.

### Result analysis

**Fig. 5:**
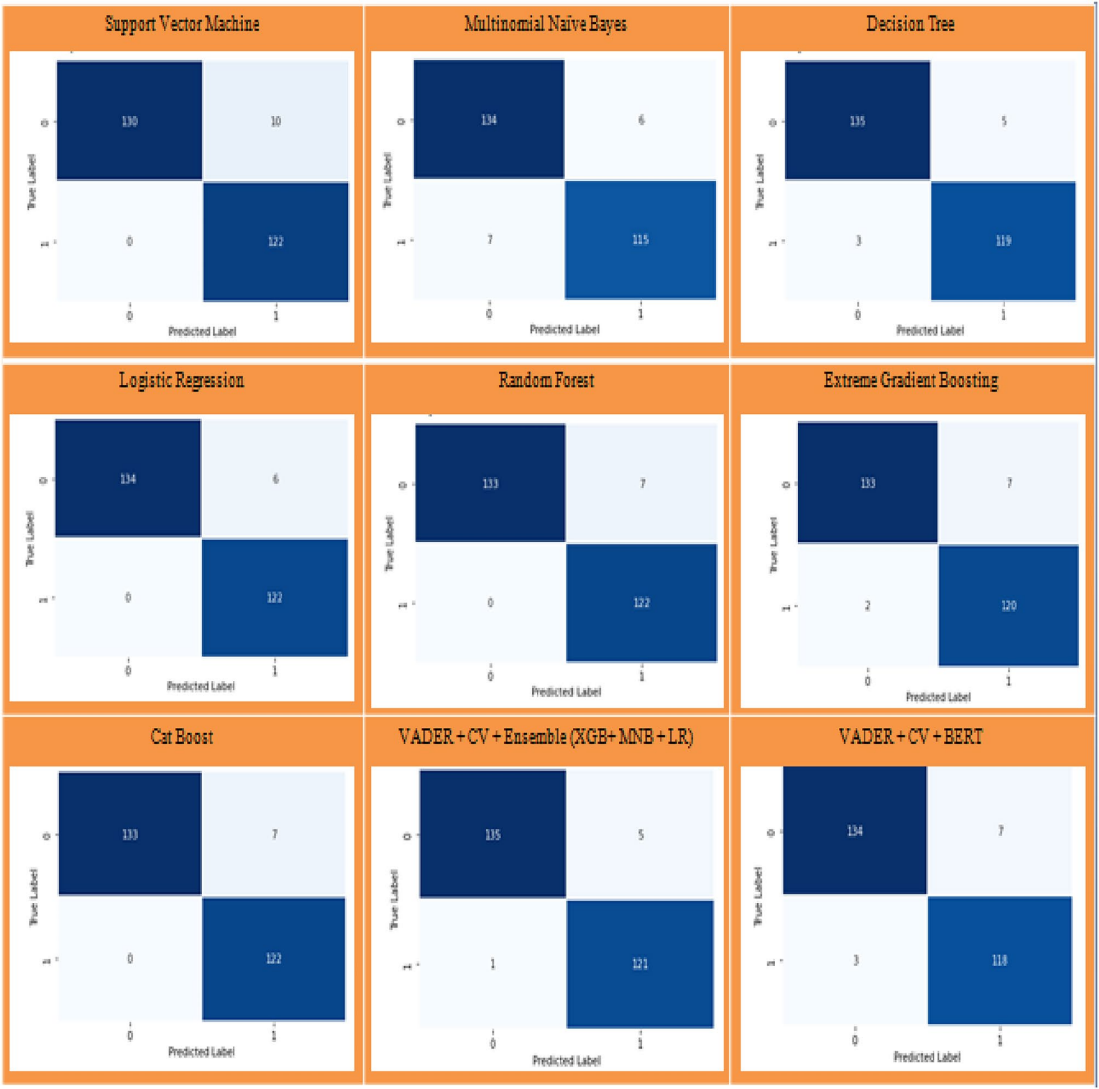
Confusion matrix of Assam flood 2022 Dataset.

The performance evaluation of the different machine learning models are configured with fixed hyper parameters to ensure fair comparison. Random Forest uses 100 trees with Gini criterion, SVM employed a linear kernel (C = 1.0), The Multinomial Naïve Bayes (MNB) classifier is configured with Laplace smoothing (alpha = 1.0), allowing effective probabilistic classification of count-based textual features. XGBoost and CatBoost are trained with 200 estimators/iterations and depth 6, and Logistic Regression used L2 regularization (C = 1.0). In addition to classical and boosting models, the BERT-base-uncased transformer is fully fine-tuned with learning rate 2e−5, batch size 16, 5 epochs to enable contextual disaster tweet classification. The system combines VADER-based labeling with Count Vectorization, followed by ensemble classifiers (RF, XGBoost, CatBoost) for structured feature learning. Additionally, a VADER + CV + BERT pipeline is implemented, where the fine-tuned BERT-base-uncased model captures contextual semantics to improve the disaster tweet classification, by updating all layers during training to adapt contextual representations for binary classification. The confusion matrix is a matrix representation that depends on the actual and predicted class of the disaster dataset and it helps to analyze the performance of disaster classification as shown in Fig. 5.

The dataset is trained and tested with different split cases such as 80:20 and 70:30, using learning models is shown in the Table 1. Some of the parameters are considered to monitor the performance of the model with respect to the dataset such as True Positive Rate (TPR), True Negative Rate (TNR), Positive Predictive Value, Negative Predictive Value (NPV), False Positive Rate (FPR), False Negative Rate (FNR), False Discovery Rate (FDR), F1-score and Accuracy (Acc).

**Table 1:** Results of different classification models of Assam flood 2022 dataset.

Classifier name	Test size (%)	F1 score	Acc
Random forest	20	0.976	0.977
	30	0.978	0.977
Multinominal Nave Bayes	20	0.975	0.977
	30	0.953	0.951
Decision tree	20	0.955	0.958
	30	0.973	0.972
Extreme gradient boosting	20	0.955	0.958
	30	0.975	0.974
Support vector machine	20	0.976	0.977
	30	0.975	0.974
Cat boost	20	0.976	0.977
	30	0.975	0.975
Logistic regression	20	0.982	0.982
	30	0.983	0.982
VADER + CV + Ensemble—[XBG + MNB + LR]	20	0.984	**0.984**
	30	0.973	0.972
VADER + CV + BERT	20	0.959	0.971
	30	0.967	0.964

VADER + CV + Ensemble—[XBG + MNB + LR] model performs well with an accuracy of 98% when compared to the other selected model.

**Fig. 6:**
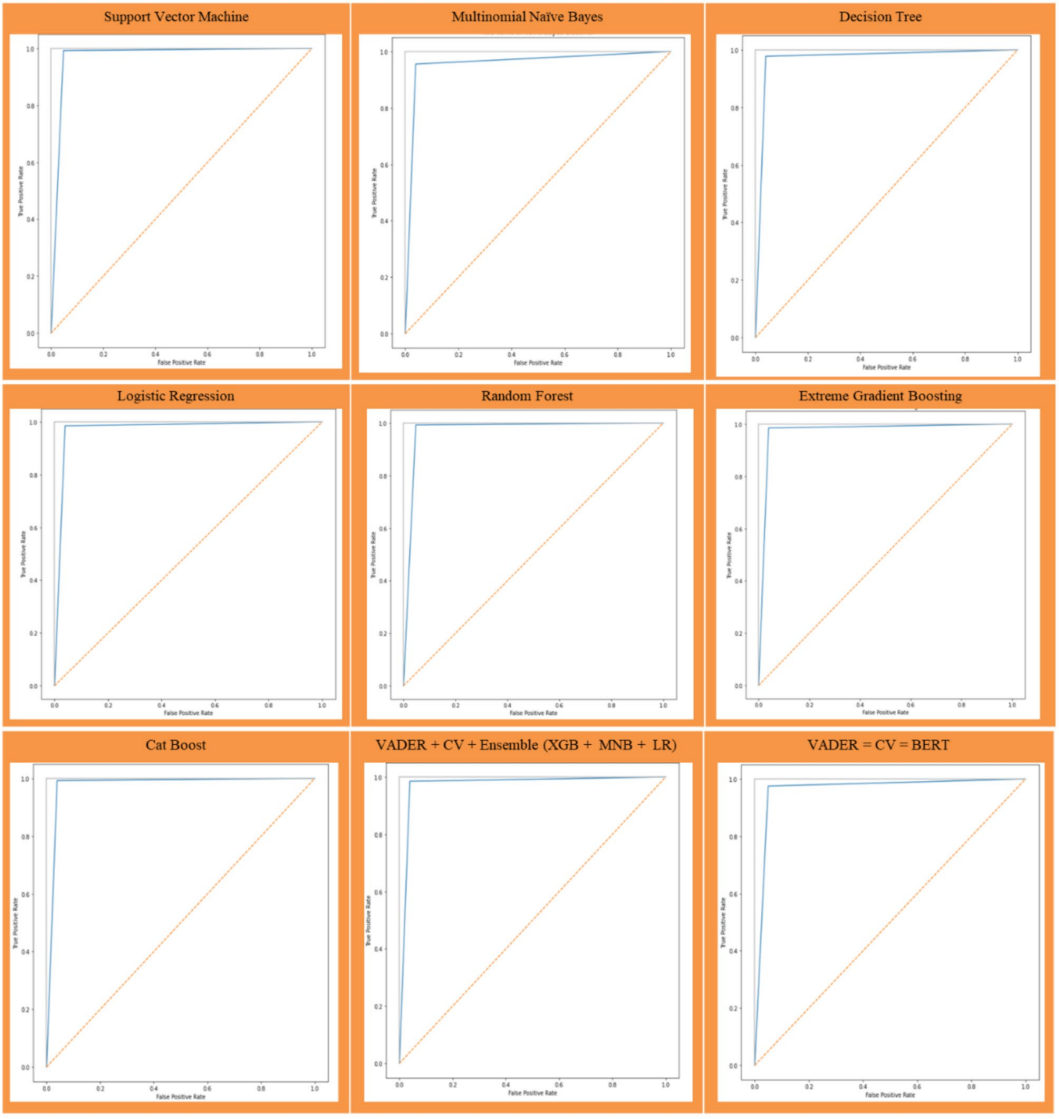
ROC curve of classifiers of Assam flood 2022 dataset.

A Receiver Operating Characteristic (ROC) is a graphical representation for evaluating the performance of the classification model with respect to the disaster dataset in which the curve focuses on both true and false positive rate obtained from the model as shown in Fig. 6. ROC curve score for SVM (0.972), MNB (0.948), DT (0.968), LR (0.976), RF (0.975), XGB (0.966), CAT (0.975), Ensemble (0.978), BERT (0.962).

**Fig. 7:**
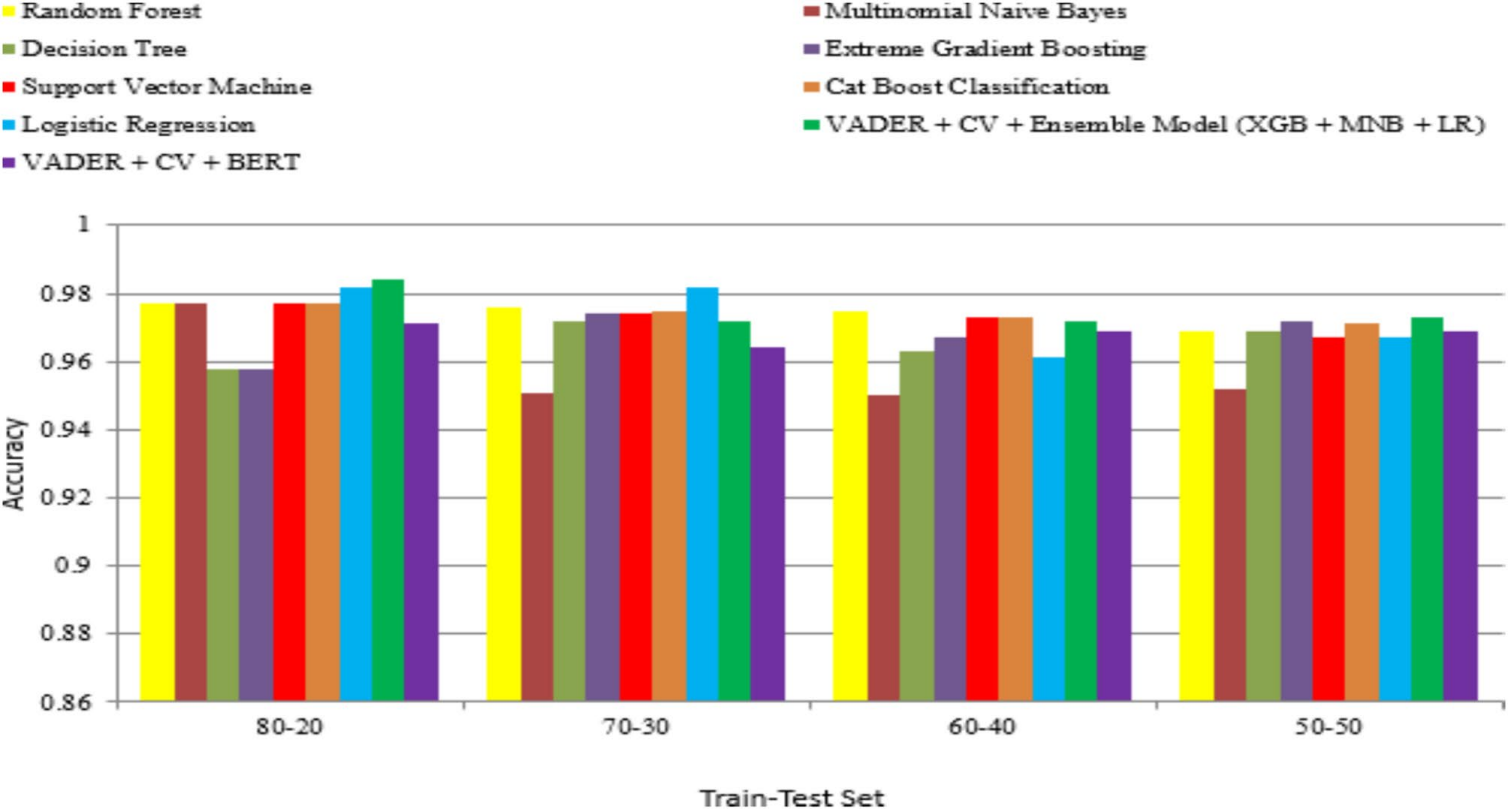
Performance comparison with classifiers.

As a result of the classification of disaster data, the 80:20 split performs better when compared to other splits. The ensemble model (XGB + MNB + LR) algorithm outperformed well compared to the other traditional machine learning model with an accuracy of 98%. BERT model produces 97% accuracy because it requires large labeled datasets and high computational resources. The ensemble model reproduces the perfect result compared to other learning classifications for classifying disaster events from the dataset as shown in Fig. 7.

After classification, 10,223 disaster tweets are considered to identify the location where 3271 tweets are geotagged tweets. The location extraction procedure is performed by using location tagger, Spacy and NER libraries helped to extract 1639 non-geotagged tweets, the remaining 5313 tweets are untagged and missed location information. The Tweets collected after the location extractions are processed again because many unrelated locations related to the event may be extracted. A new location pincode dataset is created based on the location where it comprises of location, district, state, and pincode. The SQL processing is performed by importing the sqlite3 library where the two datasets are linked to the database there by finding the common field location using the Natural Join. Finally 2007 tweets are extracted with locations that are related to the Assam State as illustrated in Table 2.

**Table 2:** Location extraction from Assam flood 2022 tweets.

Assam Flood 2022 tweets	Location extracted
RT @RashmiDVS: The man-made floods of Assam should be treated as an act of terrorism and the people arrested for the same in *Bethukandi* should be tried aâ€¦	*Bethukandi Bethukandi* *Barpeta Nagaon Silchar Bethukandi*
RT @ShefVaidya: The Assam floods were man-made! Kabul Khan, Mithu Hussain Laskar, Nazir Hussian Laskar and Ripon Khan arrested in *Bethukandi* for breachinâ€¦	
RT @AbhishBanerj: Why is this not biggest news in India? *Barpeta* is the worst affected district River Kopili continues to flow above the danger level mark in *Nagaon* district. RT @meraki_VP: Over 1.09 Lakhs of people are suffering due to Assam floods in 230 relief camp in *Silchar*	
RT @skamaraj32: The Assam Police has arrested four men for allegedly sabotaging the *Bethukandi* dyke which led to unprecedented floods thatâ€¦	

**Fig. 8:**
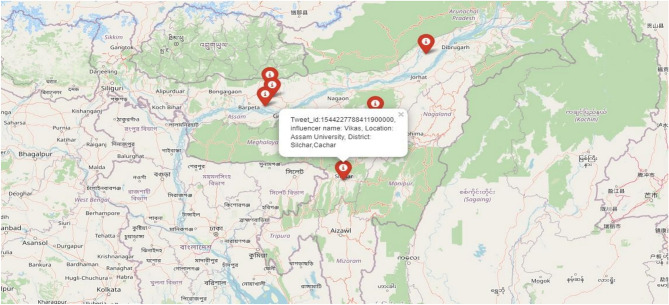
Visualization of influencers in Assam.

The louvain’s community detection algorithm detects the communication between the users, where the nodes are interconnected based on the location pincode. Totally 15,998 edges are interconnected in the graph and the modularity is identified as 0.7593. After the community is detected, the influencer in the network is identified where the centrality nodes in the communities are identified as influencers and it depends on scores. The harmonic centrality is used to detect the influencer where the algorithm obeys closeness centrality, improves the centrality scores, and solves the unconnected and undirected graph and it avoids long distance out the weight. The community’s influencer is identified and visually represented using a folium map marker where the latitude and longitude of the location are identified, to pin the location in the map. Indication of the location in the map represents whether the location is in an emergency or not and it provides the top influencer contact in the popup menu as shown in Fig. 8. So the user can share their own post and location who suffers in disaster to the influencer. Influencer spreads the information throughout the network communities, which gives better alarm for the user, rescue team, and other emergency contacts to save human life in danger or disaster.

## Conclusion and future work

The proposed a location-based bursty event detection influencer identification during crisis events from social media where LHCA system helps to detect the emergency event at the right time by finding the appropriate location of the event and diffusing the disaster information throughout the network by detecting communities and identifying influencers of disaster location helps in giving the alert message to the emergency contacts, rescue teams and providing prior information to the user who lives in that area. Assam Flood 2022 dataset is extracted from Twitter, automatic data labeling is introduced to reduce human interaction and error and then we performed implementation with traditional machine learning models, Ensemble model produces 98% accuracy and the BERT model produces 97% accuracy. The observed improvement in classification performance is primarily attributed to the integration of sentiment-aware labeling and ensemble learning, which effectively reduces noise and improves class discrimination. Additionally, the combination of multiple classifiers enhances robustness and generalization compared to individual models, resulting in superior detection accuracy. Our proposed system helps in finding disaster location information accurately by some parameters such as streets, cities, districts, states, and countries where this system can also be useful for all emergency events such as natural disasters and man-made disasters. The limited number of geo-tagged tweets and tweets with location are one of the drawbacks because in our dataset many tweets express emergency content but without the location of the tweets are considered to be useless. In the future our proposed system can be extended to support multilingual language to improve its applicability across geographic region. The real time implementation of streaming data and integrated with the mobile application to send the alert message of the disaster events to the user connected in the communities. Social media cyberbullies, fake information, and spam message on Twitter are increasing and other social media needs to be focused in the future.

## Data Availability

The dataset and the source code in this study are available upon request by contact with the corresponding author.

## Appendix

See Table 3.

**Table 3:** Results of different classification models of Assam flood 2022 dataset.

Classifier name	Test size (%)	TPR	TNR	PPV	NPV	FPR	FNR	FDR	F1 score	Acc
Random Forest	20	1.0	0.956	0.953	1.0	0.043	0.0	0.046	0.976	0.977
	30	1.0	0.952	0.957	1.0	0.047	0.0	0.042	0.978	0.977
	40	1.0	0.955	0.955	1.0	0.044	0.0	0.044	0.977	0.977
	50	1.0	0.940	0.941	1.0	0.059	0.0	0.058	0.969	0.969
Multinominal Nave Bayes	20	0.983	0.971	0.968	0.985	0.028	0.016	0.032	0.975	0.977
	30	0.955	0.947	0.950	0.952	0.052	0.044	0.049	0.953	0.951
	40	0.933	0.966	0.963	0.938	0.033	0.066	0.036	0.948	0.950
	50	0.956	0.949	0.947	0.957	0.050	0.043	0.052	0.951	0.952
Decision Tree	20	0.951	0.964	0.959	0.957	0.035	0.048	0.040	0.955	0.958
	30	0.985	0.957	0.961	0.983	0.042	0.014	0.038	0.973	0.972
	40	0.968	0.959	0.957	0.969	0.040	0.031	0.042	0.962	0.963
	50	0.984	0.955	0.954	0.984	0.044	0.015	0.045	0.969	0.969
Extreme Gradient Boosting	20	0.951	0.964	0.959	0.957	0.035	0.048	0.040	0.955	0.958
	30	0.995	0.952	0.957	0.994	0.047	0.004	0.042	0.975	0.974
	40	0.976	0.959	0.957	0.977	0.040	0.023	0.042	0.969	0.967
	50	0.996	0.949	0.949	0.996	0.050	0.003	0.050	0.972	0.972
Support Vector Machine	20	1.0	0.956	0.953	1.0	0.043	0.0	0.046	0.976	0.977
	30	1.0	0.947	0.953	1.0	0.052	0.0	0.046	0.975	0.974
	40	1.0	0.947	0.947	1.0	0.052	0.0	0.052	0.973	0.973
	50	1.0	0.937	0.938	1.0	0.062	0.0	0.061	0.968	0.967
Cat Boost	20	0.991	0.964	0.960	0.992	0.035	0.008	0.039	0.976	0.977
	30	0.995	0.952	0.957	0.994	0.047	0.004	0.042	0.975	0.975
	40	0.996	0.959	0.958	0.996	0.040	0.003	0.041	0.976	0.977
	50	1.0	0.943	0.944	1.0	0.056	0.0	0.056	0.971	0.970
Logistic Regression	20	1.0	0.971	0.968	1.0	0.028	0.0	0.031	0.982	0.982
	30	1.0	0.963	0.966	1.0	0.036	0.0	0.033	0.983	0.982
	40	0.956	0.966	0.964	0.959	0.033	0.043	0.035	0.960	0.961
	50	0.993	0.943	0.943	0.993	0.056	0.006	0.056	0.968	0.967
VADER + CV + Ensemble—[XBG + MNB + LR]	20	1.0	0.971	0.968	1.0	0.028	0.0	0.031	0.984	**0.984**
	30	0.990	0.952	0.957	0.989	0.047	0.009	0.042	0.973	0.972
	40	0.984	0.962	0.961	0.984	0.037	0.015	0.038	0.972	0.973
	50	0.996	0.946	0.946	0.996	0.053	0.003	0.053	0.971	0.970
	20	0.975	0.950	0.944	0.978	0.049	0.024	0.056	0.959	0.971
VADER + CV + BERT	30	0.979	0.952	0.946	0.980	0.047	0.022	0.053	0.967	0.964
	40	0.987	0.954	0.947	0.989	0.045	0.012	0.052	0.961	0.969
	50	0.987	0.954	0.947	0.989	0.045	0.012	0.052	0.967	0.969

VADER + CV + Ensemble—[XBG + MNB + LR] model performs well with an accuracy of 98% when compared to the other selected model.

## Abbreviations

BERT: Bi-directional encoder representations from transformers
Bi-LSTM: Bidirectional long short term memory
CBC: Cat boost classifier
CNN: Convolutional neural network
DT: Decision tree
FPR: False positive rate
FNR: False negative rate
FDR: False Discovery Rate
GloVe: Global vector for word representation
LSTM: Long short term memory
LHCA: Louvain-based harmonic centrality algorithm
LR: Logistic regression
MLM: Masked language modeling
MNB: Multinomial Naïve Bayes
NLTK: Natural language ToolKit
NPV: Negative predictive value
NSP: Next sentence prediction
PPV: Positive predictive value
ReLU: Rectified linear unit
RF: Random forest
RNN: Recurrent neural network
RoC: Receiver operating characteristic
SVM: Support vector machine
TNR: True negative rate
TPR: True positive rate
VADER: Valence aware dictionary sentiment reasoner
XGBoost: Extreme gradient boosting
